# Evaluation of a real-time PCR assay performance to detect *Mycobacterium tuberculosis*, rifampicin, and isoniazid resistance in sputum specimens: a multicenter study in two major cities of Indonesia

**DOI:** 10.3389/fmicb.2024.1372647

**Published:** 2024-05-10

**Authors:** Ida Parwati, Lidya Chaidir, Muhammad Yunus, Maya Marinda Montain, Dini Budhiarko, Siti Fatimah Selasih, Ryan Bayusantika Ristandi, Rifky Waluyajati Rachman, Raden Desy Nurhayati, Imran Pambudi, Akterono Dwi Budiyati

**Affiliations:** ^1^Department of Clinical Pathology, Faculty of Medicine, Universitas Padjadjaran/Dr. Hasan Sadikin General Hospital, Bandung, Indonesia; ^2^Department of Biomedical Sciences, Faculty of Medicine, Universitas Padjadjaran, Bandung, Indonesia; ^3^Stem Cell and Cancer Institute, PT. Kalbe Farma, Jakarta, Indonesia; ^4^Unit Pelayanan Fungsional Balai Besar Kesehatan Paru Masyarakat Bandung (UPF BBKPM), Bandung, Indonesia; ^5^West Java Provincial Health Laboratory, Bandung, Indonesia; ^6^Dr. H. A. Rotinsulu Lung Hospital, Bandung, Indonesia; ^7^Directorate General of Diseases Prevention and Control, Ministry of Health of the Republic of Indonesia, Jakarta, Indonesia

**Keywords:** *Mycobacterium tuberculosis*, real-time PCR, diagnostic, Indigen, RIF resistance, INH resistance

## Abstract

**Background:**

Tuberculosis (TB) is one of the major global health issues due to its high mortality rate, especially in low- and middle-income countries. One of the key success points of the TB eradication program is early TB diagnosis, which requires rapid and accurate diagnostic testing. This study aimed to evaluate the performance of a newly developed RT-PCR kit (Indigen MTB/DR-TB RT-PCR) in a routine TB clinical setting.

**Method:**

A multi-fluorescence RT-PCR assay was designed and developed to detect regions within *IS6110*, *rpoB, katG*, and *inhA* of the *Mycobacterium tuberculosis* (MTB) genes. Sputum specimens were obtained from suspected TB patients who visited TB healthcare facilities in two major cities of Indonesia from September 2022 to May 2023. Specimens were assessed using Indigen MTB/DR-TB RT-PCR, acid-fast bacillus (AFB) smear microscopy, MTB culture, and drug susceptibility testing (DST) methods. Fisher’s exact test (*χ*^2^) was used to analyze the Indigen performance relative to culture methods.

**Result:**

The performance of Indigen MTB/DR-TB RT-PCR to detect MTB was assessed using 610 sputum specimens obtained from suspected patients. The overall sensitivity and specificity were 94.12% (95% CI: 90.86–96.48%) and 98.32% (95% CI: 96.20–99.46%), respectively. When the analysis was performed on AFB smear-negative TB subjects (386 subjects), a lower sensitivity level was found at 78.57% (95% CI: 68.26–86.78%), while the specificity level remained similar at 98.34% (95% CI: 96.18–99.46%). The overall performance of Indigen MTB/DR-TB RT-PCR to detect MTB showed substantial agreement with the MTB culture method (kappa value 0.93). In comparison to DST, the sensitivity and specificity levels of Indigen to detect RIF resistance or INH resistance were 78.2% (95% CI: 61.8–90.2%) and 82.8% (95% CI: 64.2–94.2%), respectively, while the specificity level for both groups was at 100% (95% CI, 87.7–100%).

**Conclusion:**

Indigen MTB/DR-TB RT-PCR demonstrated reliable performance for TB molecular diagnostic testing and can be implemented in routine TB diagnostic settings.

## 1 Introduction

Tuberculosis (TB) remains the leading cause of death by a single infectious agent globally. The World Health Organization (WHO) reported that there were an estimated 10.6 million new cases of disease and 1.6 million deaths from TB in 2022 (World Health Organization, 2023). Most of the cases were contributed by low- and middle-income countries, which accounted for two-thirds of the global total cases (Chakaya et al., 2020). Of those countries, Indonesia ranked as having the second highest burden of TB behind India. Indonesia was estimated to have 969,000 TB cases in 2023 (Ministry of Health Republic Indonesia, 2020), and two-thirds of the cases were found in Java and Bali, predominantly in urban areas (Ministry of Health Republic Indonesia, 2023).

Early TB diagnosis followed by precise treatment is critical to stopping the transmission of the disease. Substantial efforts have been made by Indonesia’s national program to find TB cases in the last 5 years, including scaling up the TB diagnostic technology with a nucleic acid amplification test (NAAT)-based platform. Currently, the GeneXpert MTB/RIF assay is being used by the Indonesian government as the recommended NAAT-based diagnostic testing in the TB national program (Ministry of Health Republic Indonesia, 2023). This kit allows completely automated nucleic acid preparation and amplification in a simple and rapid TB detection process. The efforts successfully increased the case notification from 360,000 cases in 2016 to 570,000 cases in 2018. However, there were an estimated 275,000 cases in 2018 (Ministry of Health Republic Indonesia, 2020) that were still underreported, and unfortunately, the gap remains unchanged at 244,691 in 2023 (Ministry of Health Republic Indonesia, 2023).

Despite the advantages offered by GeneXpert/MTB/RIF assay, it also has shortcomings such as high cost, closed system, and requirement for imported material that need to be addressed. Given the circumstances, implementing local NAAT testing to cover the underreported gap in TB case notification is imperative. Meanwhile, there was a considerable increase in the number of laboratories equipped with advanced real-time polymerase chain reaction (RT-PCR) machines and molecular infrastructure readiness as an impact of the COVID-19 pandemic situation (Ministry of Health Republic Indonesia, 2023) waiting to be leveraged. RT-PCR itself has been accepted as a robust method for detecting *Mycobacterium tuberculosis* (MTB), making its implementation highly recommended (Espy et al., 2006; Babafemi et al., 2017).

To address the abovementioned issue, we developed an open platform RT-PCR assay that can be run on the same RT-PCR instruments that have been placed in several healthcare facilities across Indonesia, including labs that use COVID-19 diagnostic testing facilities. The assay was designed to detect the region within the insertion sequence of the *IS6110*, *rpoB*, *katG*, and *inhA* (*C15T*) genes of MTB. The *IS6110* region has been widely used as a target for MTB detection due to its relatively high sensitivity related to its presence in multiple copies (ranging from 1 to 25 copies depending on bacterial strains) within the MTB genome (Eisenach et al., 1990; Peng et al., 2017). Meanwhile, mutations within the 81-base pair core region of rpoB, corresponding to codons 507–533, are associated with resistance to rifampicin (RIF), the first-line and most common antibiotic for treating TB. Within this *rpoB* hot spot region, we targeted two of the most prevalent mutations, which are mutations at codons 531 and 526 (Ramaswamy and Musser, 1998; Peng et al., 2017). On the other hand, mutations at codon 315 of *katG* and codon 15 of *Inh* genes were known to be the most prevalent hot spot mutations related to resistance to isoniazid (INH), the other first-line drug against TB worldwide (Ramaswamy and Musser, 1998). Harnessing the ability of Indigen MTB/DR-TB RT-PCR to detect MTB while at the same time detecting MTB RIF-resistant and INH-resistant strains, as well as common NTM infections, would be the strongest value proposition of the kit for leveraging TB capacity testing in Indonesia.

We evaluated the Indigen MTB/DR-TB RT-PCR assay performance by conducting a clinical validation study in Jakarta and Bandung, the two major cities in Indonesia with more than 250 TB incidences per 100,000 (Alisjahbana et al., 2021; Ministry of Health Republic Indonesia, 2023). In order to assess the use of Indigen in the routine clinical TB setting, the protocol was applied in such a way that the workflow and diagnostic methodologies were maintained as recommended by national TB guidelines.

## 2 Methods

### 2.1 Study design, clinical specimens, and ethics consideration

The clinical performance study was designed as a non-interventional and multicenter study from September 2022 to May 2023. Sputum specimens were obtained from suspected TB patients who visited primary healthcare facilities in Bandung, i.e., UPF BBKPM and Dr. H. A. Rotinsulu Pulmonary Hospital, or from routine sputum specimens that were sent to the national referral TB diagnostic laboratory at Bandung (West Java Provincial Health Laboratory/WJPHL) and Jakarta (BBLK Jakarta). Only raw sputum with a minimum volume of 4 mL was included in this study. In addition to these, we assessed archived specimen leftovers from the Research Center for Care and Control of Infectious Disease (RC3ID) Universitas Padjadjaran Bandung. Acid-fast bacilli (AFB) smears and culture assays were performed at the respective laboratories, whereas Indigen RT-PCR was performed at Stem Cell and Cancer Institute (SCI) Jakarta, WJPHL in Jakarta, and BBLK in Jakarta. Each sputum sample represented one subject. In order to obtain sufficient power to determine sensitivity and specificity to the 95% confidence interval level for this study, we were targeting to get as few as 300 MTB positive and 300 MTB negative sputum samples based on MTB culture as reference method (Mattocks et al., 2010). All the testing methods were performed blindly between methods and between sites to obtain accountable results.

This study was approved by the Ethics Committee at the Universitas Padjadjaran Bandung, under ethical approval number 876/UN6.KEP/EC/2022. The specimen collection process was a part of the TB-routine clinical diagnostic setting; therefore, informed consent was not required.

### 2.2 Acid-fast bacilli (AFB) smear microscopy and culture

The presence of AFB was assessed using the Ziehl–Neelsen staining method. It was performed directly in unprocessed sputum at each laboratory site, and the remaining samples were then processed for MTB culture and RT-PCR procedure. In brief, the sputum samples were decontaminated and homogenized using NaLC-4% NaOH according to the protocol recommended by the Ministry of Health for TB diagnostic laboratory guidelines (Ministry of Health Republic of Indonesia, 2021). Following sediment resuspension with phosphate-buffered saline (PBS), the suspension was inoculated into a Lowenstein–Jensen (LJ) tube (0.5 mL) or inoculated into a liquid Bactec mycobacteria growth indicator tube (MGIT) 960 (0.2 mL) (Becton Dickinson, Sparks, MD, USA) at 37°C or at 35°C for 8 weeks, respectively (Vinuesa et al., 2018; Ministry of Health Republic of Indonesia, 2021). Every positive culture was subjected to Ziehl–Neelsen staining to confirm the presence of AFB and cord formation and to exclude contamination. Drug susceptibility testing (DST) was conducted to determine the phenotype of mycobacterial strains against RIF and INH and used as the gold standard reference for Indigen DR/MDR-TB RT-PCR performance to detect MTB RIF- or INH-resistance strains (Ministry of Health Republic of Indonesia, 2021).

### 2.3 Indigen MTB/TB-DR RT-PCR assay

The Indigen MTB/TB-DR RT-PCR assay was designed to detect key mutations in four gene regions associated with RIF/INH resistance as well as MTB presence. Five dually labeled probes and four pairs of primers have been designed based on (Peng et al., 2017, Supplementary Table 1). Since most of the RT-PCR machines placed in laboratories are available in a 4-channel model, we designed the assay to have two PCR reactions for each sample. Four fluorophores (FAM, HEX, ROX, and Cy5) are attached at the 5′ end of probes, which allows the detection of the target biomarkers in a single PCR reaction simultaneously. In brief, 750 μL of raw sputum or decontaminated or sediment sputum was mixed with an equal volume of lysis buffers in a microcentrifuge tube. The mixture was incubated at room temperature for 30 min with intermittent agitation. Following centrifugation at 14,000 rpm for 3 min, the supernatant was discarded and replaced by 1 mL of saline buffer. The mixture was centrifuged at 14,000 rpm for 3 min, and the supernatant was discarded. A measure of 200 μL of Chelex® solution was added, followed by incubation at 100°C and then centrifugation at 14,000 rpm for 10 min. The supernatant was transferred to a new microcentrifuge tube and used directly as a PCR template or stored at -20°C. All reagents for the DNA extraction procedure were provided in the kit. PCR mixture contained 2 μL of 10 × PCR buffer, 1.6 μL of dNTP (each 2.5 μM), 0.8 μL of MgCl2 (25 mM), 0.2 μL of Taq-polymerase (5 U/ul), 2 μL of primers and probes extracted DNA template of 1 μL, and Milli-Q H_2_O to make up the whole PCR mixture volume to 20 μL for each PCR reaction. The PCR cycle was run in a Rotor-Gene Q real-time rotary analyzer (Qiagen, Hilden, Germany) or in a LightCycler instrument (Roche). Cycling parameters consist of denaturation denaturation at 95°C for 15 min, followed by 45 cycles of amplification at 95°C for 15 s, and elongation at 60°C for 1 min. An internal extraction control (IC) for each sample, as well as a negative and positive control for each run, were also included.

Result interpretation was performed based on a simple algorithm. First, the positive signal at the IS6110 probe represents MTB positivity. Second, signals of the other four drug-resistant probes were investigated. When all RIF and INH probes show a negative signal, it is classified as MTB drug-susceptible. On the other hand, when the RIF probes show positive signals, either at S531L and/or H526Y, the sample is classified as RIF resistance. Similarly, the sample is classified as INH resistance when katG and/or inhA probes are positive. Drug-susceptible TB patients can be treated with first-line TB therapy, while TB patients with RIF-mono resistance or INH-mono resistance are considered to receive first-line anti-TB drugs, except RIF or INH, respectively. In addition to this, when both RIF and INH resistance are present, the TB patients are classified as multi-drug resistant and considered to receive a combination of second-line anti-TB drugs (Ministry of Health Republic of Indonesia, 2021).

### 2.4 Limit of detection and analytical specificity of Indigen MTB/DR-TB RT-PCR

We prepared solution of each bacterial isolate (MTB HRa37, MTB with rpoB S531L, MTB with rpoB H526Y, MTB with katG S315T, MTB with inhA C15T, and *M. smegmatis*) corresponding to McFarland standard no. 1. The limit of detection (LoD) was initially performed by 10-fold dilution of each bacterial isolate in triplicates. The expected LoD was determined, and all replicates were positive. The LoD was then confirmed by repeating the assay using five different serial dilutions of concentration covering the expected range of LoD, which was performed in 20 replicates for each dilution. Final LoD was determined based on the lowest colony forming unit per milliliter (CFU/mL) at ≥95% positivity rate of each bacterial isolate.

The analytical specificity of the probe sequence of *IS6110* was conducted *in silico*. Sequence alignment was performed against PCR products and surrounding sequences of 35 types of mycobacteria, 66 types of non-mycobacteria bacteria, 12 types of fungi, and 14 types of viruses. All sequences were downloaded from https://blast.ncbi.nlm.nih.gov/Blast.cgi. If the designed probes have less than 80% homology in terms of the matched base pair amount with target organisms, potential cross-reactivity may be prevented.

### 2.5 Sequencing

Bidirectional sequencing was performed on samples with discrepant results using the BigDye Terminator Cycle Sequencing kit, according to the manufacturer’s recommendations in a 3130xl Genetic Analyzer (Applied Biosystems). Traces were analyzed with BioEdit v7 2.5.

### 2.6 Statistical analysis

Fisher’s exact test (*χ*^2^) was used to analyze the sensitivity and specificity level of Indigen MTB RT-PCR assay on sputum samples compared to culture, which is considered the TB gold standard method for TB diagnosis. The kappa test was used to measure agreement between the two methods (MedCalc Software Ltd, 2023).

## 3 Results

### 3.1 Sputum samples and phenotypic analysis based on AFB smears and MTB culture

A total of 610 sputum samples from 610 TB-suspected subjects were collected from our five study sites. Most of our prospectively collected samples were collected from WJPHL (305 samples), followed by UPF BBKPM, BBLK Jakarta, and Dr. H. A. Rotinsulu Pulmonary Hospital with 105, 89, and 22 samples, respectively. Meanwhile, our archived samples from RC3ID Laboratory were 89 samples. Overall, our cohort samples consist of 306 MTB culture-positive and 304 MTB culture-negative samples. Stratification of samples based on the AFB smear method resulted in 224 smear-positive samples and 386 smear-negative samples, as described in Table 1.

**Table 1:** Performance of Indigen MTB/DR-TB RT-PCR for detecting MTB in all sputum samples and in samples stratified according to smear results.

AFB smear microscopy result	Indigen MTB/DR-TB	MTB culture positive	MTB culture negative	Sensitivity, % (95% CI)	Specificity, % (95% CI)	PPV (95% CI)	NPV (95% CI)	Accuracy (95% CI)	*ƙ*-value ± SE
Overall (*N* = 610)	MTB positive	288	5	94.1 (90.9–96.5)	98.4 (96.2–99.5)	98.3 (96.0–99.3)	94.4 (91.4–96.3)	96.2 (94.4–97.6)	0.9 ± 0.02 0.9–1
	MTB negative	18	299						
Positive (*N* = 224)	MTB positive	222	0	100 (98.4–100)	100 (15.8–100)	100 (98.4–100)	100 (15.8–100)	100 (98.4–100)	1 ± 0 1–1
	MTB negative	0	2						
Negative (*N* = 386)	MTB positive	66	5	78.6 (68.3–86.8)	98.3 (96.2–99.5)	93 (84.6–97)	94.3 (91.6–96.1)	94 (91.2–96.2)	0.8 ± 0.04 0.7 ± 0.9
	MTB negative	18	297						

PPV, positive predictive value; NPV, negative predictive value.

### 3.2 Limit of detection and analytical specificity of Indigen MTB/DR-TB RT-PCR

The LoD of Indigen MTB/TB DR RT-PCR is 400 CFU/mL for the IS6110 detection, 6,000 CFU/mL for the *rpoB* S531L and *rpoB* H526Ydetection, 400 CFU/mL for the *katG* S315T detection, and 6,000 CFU/mL for the *inhA* C15T detection (Supplementary Data 1). Analytical specificity test demonstrated that the *IS6110* probe has no homology with greater or equal to 80% against listed bacteria (mycobacteria and non-mycobacteria), fungi, and viruses (Supplementary Data 2).

### 3.3 Performance of Indigen MTB/DR-TB RT-PCR on detecting MTB in sputum

The results of culture, AFB smear microscopy, and Indigen MTB/DR-TB RT-PCR are given in Table 1. Compared to culture, the overall sensitivity and specificity of Indigen MTB/DR-TB RT-PCR in detecting MTB were 94.12% (95% CI 90.86–96.48) and 98.36% (95% CI 96.20–99.46), respectively. The positive predictive value (PPV) and negative predictive value (NPV) were 98.29% (95% CI 96.02–99.28) and 94.41% (95% CI 91.39–96.30). However, when the analysis was stratified based on AFB smear-negative samples (386 samples), a lower sensitivity level was found at 78.57% (95% CI, 68.26–86.78%), while the specificity remained similar at 98.34% (95% CI, 96.18–99.46%). On the other hand, the PPV and NPV levels for these AFB smear-negative samples were 92.96% (95% CI 84.60–96.94) and 94.29% (95% CI 91.63–96.13). Corresponding values for sensitivity, specificity, PPV, and NPV of the other 224 AFB smear-positive samples were 100 (95% CI 98.35–100.00), 100 (95% CI 15.8–100.00), 100 (95% CI 98.35–100.00), and 100 (95% CI 15.81–100.00), respectively. Overall agreement between culture and Indigen MTB/DR-TB RT-PCR was 96.23% (95% CI 94.40–97.60).

### 3.4 Performance of Indigen MTB/DR-TB RT-PCR on detecting MTB RIF/INH resistance in sputum

We assessed the Indigen MTB/DR-TB RT-PCR ability to detect drug-resistant (MTB DR) on 65 samples that already had DST results and sufficient volume for RT-PCR. Of these samples, 28 were MTB drug-susceptible, and the remaining 37 samples were MTB DR samples. Based on the DST result, the MTB DR samples mainly consisted of 78.4% (29/37) double RIF- and INH-resistant and 21.6% (8/37) RIF-mono-resistant. Our result showed that the sensitivity and specificity levels of Indigen MTB/DR-TB RT-PCR to detect RIF resistance and INH resistance were 78.2% (95% CI, 61.8–90.2%) and 82.8% (95% CI, 64.2–94.2%), respectively, while the specificity level for both groups is at 100% (95% CI, 87.7–100%) (Table 2).

**Table 2:** Performance of Indigen MTB/DR-TB RT-PCR based on rifampicin and isoniazid phenotypic susceptibility testing.

Target drug	Indigen MTB/DR-TB results	Phenotypic DST testing results (*n* = 65)		Sensitivity, % (95% CI)	Specificity, % (95% CI)	PPV (95% CI)	NPV (95% CI)	Accuracy (95% CI)	*ƙ*-value
		Resistant	Susceptible						
Rifampicin	Resistant	29	0	78.4 (61.8–90.2)	100 (87.7–100)	100 (88.1–100)	77.8 (65.5–86.6)	87.7 (77.2–94.5)	0.8 ± 0.08 0.6–0.9
	Susceptible	8	28						
Isoniazid	Resistant	24	0	82.8 (64.2–94.2)	100 (90.3–100)	100 (85.8–100)	87.8 (76.4–94.1)	92.3 (83–97.5)	0.8 ± 0.07 0.7–1
	Susceptible	5	36						

DR, drug resistance to RIF- or INH; PPV, positive predictive value; NPV, negative predictive value.

We found eight samples with false-susceptible results for RIF resistance. These samples contained MTB with mutations other than the targets of Indigen TB DR/MDR RT-PCR. Of these samples, three contained MTB with single amino acid substitutions at D526Y/H526D/H526H, while five contained multiple amino acid substitutions, including G523G, D516Y, H526N, H526Q, L521L, L524L, L530L, P520P, R528R, R529R, S522A, S522G, S531F, S531N, and 518-Del (Table 3). Meanwhile, we found five false-susceptible samples for INH resistance. Of these samples, four were confirmed susceptible, while one sample contained amino acid substitution at *katG* (S315T) (Table 3).

**Table 3:** Sanger sequencing analysis for samples with discordant susceptibility results.

ID	Indigen MTB/DR-TB RT-PCR	Phenotypic DST		RIF sequencing results
		RIF	INH	
2400214	TB+, INH Resistance	Resistant	Resistant	P520P, L521L, S522A, G523G, L524L, R529R
2400297	TB+, INH resistance	Resistant	Resistant	P520P, L521L, S522A, G523G, R528R
2400284	TB+, INH resistance	Resistant	Resistant	P520P, L521L, S522G, L530L, S531F
2400301	TB+, INH resistance	Resistant	Resistant	D516Y
2400184	TB+, INH resistance	Resistant	Resistant	L521L, G523G, L524L, R528R, R529R
2400182	TB+, INH resistance	Resistant	Resistant	H526D
2400197	TB+, INH resistance	Resistant	Resistant	P520P, G523G, R529R
2400185	TB+, INH resistance	Resistant	Resistant	518_Del, H526N
ID	Indigen MTB/DR-TB RT-PCR	Phenotypic DST		INH sequencing result
		RIF	INH	
2400189	TB+, RIF resistance	Resistant	Resistant	Wild type
2400208	TB+, RIF resistance	Resistant	Resistant	Wild type
2400162	TB+, RIF resistance	Resistant	Resistant	Wild type
2400210	TB+, RIF resistance	Resistant	Resistant	Wild type
2400214	TB+, RIF-sensitive, INH-sensitive	Resistant	Resistant	S315T

## 4 Discussion

Early TB diagnosis followed by precise treatment is critical to stopping the transmission of TB disease. Despite a significant increase in NAAT implementation for TB diagnosis such as GeneXpert MTB/RIF in Indonesia, considerable underreporting of TB cases remains to be addressed (Ministry of Health Republic Indonesia, 2020, 2023). In this study, we demonstrated the clinical performance of a newly developed TB NAAT, the Indigen DR/MDR RT-PCR, on 610 sputum samples obtained from 610 TB-suspected patients. This kit was designed as an open-system RT-PCR that can be implemented in any molecular laboratory equipped with an RT-PCR machine. The clinical performance of Indigen DR/MDR RT-PCR on detecting MTB, RIF, and INH resistance was almost in perfect agreement with MTB culture and DST, as shown by a kappa value of ≥0.8 in all analyses: overall samples, stratified based on AFB smear microscopy results, DR samples, and MDR samples. The findings support the potential use of Indigen MTB/DR-TB RT-PCR as an early TB diagnostic in clinical laboratory settings.

The gold standard technique for TB diagnostics is culture; however, the process requires 2–8 weeks to complete. NAAT-based testing, such as RT-PCR, has been widely accepted for TB diagnosis as it offers great robustness and a shorter turnaround time. However, to be accepted as a TB diagnosis NAAT, an assay should demonstrate high reliability, sensitivity, specificity, and accessibility levels. To achieve those requirements, one of the most determining factors is primers specific to target sequences within the MTB genome. Most TB PCR-based assays target *IS6110* to detect MTB in pulmonary and extra-pulmonary specimens (Noordhoek et al., 1994; Lee et al., 2014; Araya et al., 2021). It has been reported that the sensitivity and specificity of *IS6110* sequence-based PCR for the detection of MTB differ among trials, ranging from 50 to 90% and 60 to 100%, respectively, compared to culture (Noordhoek et al., 1994). Despite this wide range of performance, our study demonstrated that the overall sensitivity and specificity of the Indigen MTB/DR-TB RT-PCR assay to detect MTB in sputum were 94.1 and 98.1%, respectively, in comparison to the standard TB culture method, along with a kappa value of ≥0.9. Concordant with our results, studies on the performance of a commercial IS6110 sequence-based PCR assay, Abbot Real-Time RIF/INH, reported a sensitivity level of 92.4% (Araya et al., 2021), while another meta-analysis study confirmed that the assay had sensitivity and specificity levels of 96 and 97%, respectively (Wang et al., 2019). Moreover, another comparison study between the *IS6110*-Taq Man-based PCR assay and GeneXpert RIF/INH reported that *IS6110*-Taq Man-based had better sensitivity (84% vs. 79%, respectively, 19). All these findings support that multicopy target *IS6110*, which is present at 10 to 15 copies in most genomes of MTB, contributes to the better sensitivity of the TB PCR-based assay (Eisenach et al., 1990; Araya et al., 2021).

Approximately 17% of TB transmission was attributable to the presence of AFB smear-negative, culture-positive TB patients. It was estimated that 20 to 50% of pulmonary TB patients are AFB smear negative (Sarmiento et al., 2003). The TB diagnostic assay should overcome this low-load AFB specimen to deliver good performance, allowing clinicians to deliver early correct treatment to patients and prevent disease transmission. The proportion of AFB smear-negative among culture-positive TB samples in our cohort was 84/310 (27.1%), and Indigen MTB/DR-TB was able to detect 66 of them (78.6% sensitivity) with a kappa value of ≥0.8. In line with our results, a meta-analysis approach on six studies by Sarmiento et al. (2003) reported that the sensitivity of PCR-based testing for the diagnosis of MTB in AFB smear-negative sputum was 70% compared to the standard culture method. Contrarily, the same study also reported that the sensitivity ranged between 32 and 92% depending on the type of sample and the type of study design. Another study demonstrated that the performance of an IS6110 sequence-based PCR on AFB smear-negative sputum was 64.5% (Lodha et al., 2022). In addition, another previous study revealed that IS6110-TaqMan assay performed better sensitivity than GeneXpert MTB/RIF on AFB smear-negative sputum (69 vs. 48%, 19), while another study demonstrated GeneXpert MTB/RIF had a sensitivity level of 73.1% compared to culture (Lombardi et al., 2017).

Several factors might contribute to the variability in the sensitivity of *IS6110* sequence-based PCR found in different studies. Regarding this, apart from the target sequence used, the diagnosis performance might depend on the efficiency of the DNA extraction process. The Indigen MTB/DR-TB RT-PCR involves simple steps, including a lysis procedure, a heating process, followed by sedimentation through centrifugation. Although the process requires more time to complete, these manual steps could be more reliable for AFB smear-negative samples due to their better efficiency in eliminating the inhibiting substances and allowing optimal bacterial lysis compared to the cartridge-based GeneXpert MTB/RIF system (Kolia-Diafouka et al., 2018). Furthermore, a study by Tan et al. (1997) showed that a simple DNA release method did not seem to impair the sensitivity of PCR assays. This simple procedure could be easily integrated into the routine schedule of a mycobacteriology laboratory without changing the existing TB workflow. In general, the abovementioned factors might support the Indigen MTB/DR-TB’s good performance in detecting MTB in this study.

Indigen MTB/DR-TB RT-PCR discrepant results for MTB detection were observed in 18 (5.88%) false-negative MTB samples. As expected, they were included in the AFB smear-negative samples. Of these samples, 13 showed a late cycle threshold between 35 and 37 cycles, while the remaining gave an undetectable amplification signal. Adjusting the Ct threshold value up to 40 cycles would have eliminated 13 of the 18 samples; however, this would simultaneously decrease the specificity level. Since the Indigen MTB/DR-TB assay demonstrated specificity above 97% in all analysis categories, the assay was considered to have optimum performance.

The prevalence of drug-resistant MTB strains can vary between and within countries. According to the WHO’s Global Tuberculosis Report, the prevalence of RIF-resistant TB in new cases was estimated to be approximately 3.4% in 2020 (World Health Organization, 2023), while a lower prevalence of 1.3% (12,531/969,000) was reported by the government of Indonesia in 2023 (Ministry of Health Republic Indonesia, 2023). INH resistance is often closely associated with RIF resistance, and their co-occurrence is commonly defined as MDR (Armand et al., 2011; World Health Organization, 2023). Detecting resistance mutations in primary samples enables the immediate initiation of the correct treatment. In order to support early diagnosis in DR/MDR cases, Indigen MTB/DR-TB RT-PCR was designed to detect two frequent mutations related to RIF resistance and two frequent mutations related to INH resistance. In this study, its performance was compared to DST on 65 samples. In terms of detecting RIF resistance, Indigen was able to detect 29 of 37 RIF-resistant samples (78.4% sensitivity) and correctly detect 28 MTB-susceptible samples (100% specificity). The performance was considered lower than previously established TB assays, such as Abbot Real-Time RIF/INH (Wang et al., 2019) or GeneXpert MTB/RIF (Boehme et al., 2010). The discrepant results for false RIF-susceptible were observed in 22% (8/37) of DR/MDR cases, and these were mainly because of mutations within 81-rpoB hotspot regions that were out of the target range of Indigen DR/MDR RT-PCR (Table 3). In addition to this, Indigen was able to detect 24 of 29 INH-resistant samples (82.8% sensitivity) and correctly detect 36 INH-susceptible samples (100% specificity). The sensitivity was lower than Abbot Real-Time RIF/INH^18^ but higher than other PCR assays that also cover INH-resistance mutations (FT-MTBDR; Hain Lifescience; 26).

Four of five false-susceptible INH discrepant results were confirmed to harbor wild-type sequences at *katG* and *inhA* (C15T). The reason for the lack of detection based on Indigen was unknown because Sanger sequencing results support our findings. One sample harbored a mutation at the *katG* (S315T) sequence that was actually detected nearly above our Ct value threshold. In this case, adjusting the Ct value threshold up to 40 might increase the sensitivity level; however, this would require another DR study to confirm the validity of the adjusted Ct value threshold and would be considered for our future research.

There are limitations to the current study that should be considered. First, a relatively small number of samples were included for DR-TB analysis. This may have limited the statistical power of the assessment of the Indigen MTB/DR-TB RT-PCR analysis. Second, we included archived, decontaminated frozen samples in this study. Therefore, the length of storage time might affect the quality of isolated nucleic acids, thus lowering the quantity of targeted DNA. Finally, we are fully aware of the limited coverage of the MTB RIF-resistance region of Indigen MTB/DR-TB RT-PCR; however, the kit is also equipped with probes targeting frequent INH-resistant MTB, which enable MDR detection in samples simultaneously.

## 5 Conclusion

The Indigen MTB/DR-TB RT-PCR assay evaluated in this study demonstrated reliable sensitivity compared to the gold standard method of culture. The Indigen MTB/DR-TB RT-PCR detects RIF resistance as well as INH resistance, providing clinicians with more resistance information to deliver prompt treatment early. Further studies are required to evaluate the performance of this kit in more clinical sputum from suspected DR-TB patients.

## Data availability statement

The original contributions presented in the study are included in the article/Supplementary material, further inquiries can be directed to the corresponding author.

## Author contributions

IPar: Conceptualization, Resources, Writing – review & editing.LC: Conceptualization, Resources, Supervision, Writing – review & editing. MY: Conceptualization, Data Curation, Formal analysis, Investigation, Writing – original draft, Writing – review & editing. MM: Resources, Supervision, Writing – review & editing. DB: Conceptualization, Data Curation, Formal analysis, Writing – original draft, Writing – review & editing. SS: Conceptualization, Data Curation, Writing – original draft, Writing - review & editing. RRi: Resources, Supervision, Writing – review & editing. RRa: Resources, Supervision, Writing – review & editing. RN: Resources, Supervision, Writing – review & editing. IPam: Resources, Supervision, Writing – review & editing. AB: Conceptualization, Supervision, Data Curation, Formal analysis, Writing – original draft, Writing – review & editing.

## Ethics statement

The studies involving humans were approved by the Ethics Committee at Universitas Padjadjaran Bandung, ethical approval number 876/UN6.KEP/EC/2022. The studies were conducted in accordance with the local legislation and institutional requirements. The participants provided their written informed consent to participate in this study.

## References

[ref1] Alisjahbana B. Koesoemadinata R. C. Hadisoemarto P. F. Lestari B. W. Hartati S. Chaidir L.. (2021). Are neighbourhoods of tuberculosis cases a high-risk population for active intervention? A protocol for tuberculosis active case finding. PLoS One 16:e0256043. doi: 10.1371/journal.pone.0256043, PMID: 34388190 PMC8362935

[ref2] Araya B. T. Ali K. E. Geleta D. A. Tekele S. G. Tulu K. D. (2021). Performance of the Abbott RealTime MTB and RIF/INH resistance assays for the detection of *mycobacterium tuberculosis* and resistance markers in sputum specimens. PLoS One 16:e0251602. doi: 10.1371/journal.pone.0251602, PMID: 33979395 PMC8115802

[ref3] Armand S. Vanhuls P. Delcroix G. Courcol R. Lemaître N. (2011). Comparison of the Xpert MTB/RIF test with an IS6110-TaqMan real-time PCR assay for direct detection of *Mycobacterium tuberculosis* in respiratory and nonrespiratory specimens. J. Clin. Microbiol. 49, 1772–1776. doi: 10.1128/JCM.02157-10, PMID: 21411592 PMC3122654

[ref4] Babafemi E. O. Cherian B. P. Banting L. Mills G. A. Ngianga K. (2017). Effectiveness of real-time polymerase chain reaction assay for the detection of *Mycobacterium tuberculosis* in pathological samples: a systematic review and meta-analysis. Syst. Rev. 6:215. doi: 10.1186/s13643-017-0608-2, PMID: 29070061 PMC5657121

[ref5] Boehme C. C. Nabeta P. Hillemann D. Nicol M. P. Shenai S. Krapp F.. (2010). Rapid molecular detection of tuberculosis and rifampin resistance. N. Engl. J. Med. 363, 1005–1015. doi: 10.1056/NEJMoa0907847, PMID: 20825313 PMC2947799

[ref6] Chakaya J. Khan M. Ntoumi F. Aklillu E. Fatima R. Mwaba P.. (2020). Reflections on the global TB burden, treatment and prevention efforts. Int. J. Infect. Dis. 113 Suppl 1, S7–S12. doi: 10.1016/j.ijid.2021.02.107, PMID: 33716195 PMC8433257

[ref7] Eisenach K. D. Cave M. D. Bates J. H. Crawford J. T. (1990). Polymerase chain reaction amplification of a repetitive DNA sequence specific for *Mycobacterium tuberculosis*. J. Infect. Dis. 161, 977–981. doi: 10.1093/infdis/161.5.977 2109022

[ref8] Espy M. J. Uhl J. R. Sloan L. M. Buckwalter S. P. Jones M. F. Vetter E. A.. (2006). Real-time PCR in clinical microbiology: applications for routine laboratory testing. Clin. Microbiol. Rev. 19, 165–256. doi: 10.1128/CMR.19.1.165-256, PMID: 16418529 PMC1360278

[ref9] Kolia-Diafouka P. Godreuil S. Bourdin A. Carrère-Kremer S. Kremer L. Van de Perre P.. (2018). Optimized lysis-extraction method combined with IS6110-amplification for detection of *Mycobacterium tuberculosis* in Paucibacillary sputum specimens. Front. Microbiol. 9:9. doi: 10.3389/fmicb.2018.02224 30319564 PMC6167964

[ref10] Lee H. Park K. G. Lee G. Park J. Park Y. G. Park Y. J. (2014). Assessment of the quantitative ability of AdvanSure TB/NTM real-time PCR in respiratory specimens by comparison with phenotypic methods. Ann. Lab. Med. 34, 51–55. doi: 10.3343/alm.2014.34.1.51, PMID: 24422196 PMC3885773

[ref11] Lodha L. Mudliar S. R. Singh J. Maurya A. Khurana A. K. Khadanga S.. (2022). Diagnostic performance of multiplex PCR for detection of *Mycobacterium tuberculosis* complex in presumptive pulmonary tuberculosis patients and its utility in smear negative specimens. J. Lab. Physicians. 14, 403–411. doi: 10.1055/s-0042-1757231, PMID: 36531543 PMC9750742

[ref12] Lombardi G. Di Gregori V. Girometti N. Tadolini M. Bisognin F. Dal Monte P. (2017). Diagnosis of smear-negative tuberculosis is greatly improved by Xpert MTB/RIF. PLoS One 12:e0176186. doi: 10.1371/journal.pone.0176186, PMID: 28430807 PMC5400262

[ref13] Mattocks C. J. Morris M. A. Matthijs G. Swinnen E. Corveleyn A. Dequeker E.. (2010). A standardized framework for the validation and verification of clinical molecular genetic tests. Eur. J. Hum. Genet. 18, 1276–1288. doi: 10.1038/ejhg.2010.101, PMID: 20664632 PMC3002854

[ref14] MedCalc Software Ltd. Diagnostic test evaluation calculator. (2023). Available at: https://www.medcalc.org/calc/diagnostic_test.php (Version 22.014); Accessed October 18, 2023).

[ref15] Ministry of Health Republic Indonesia (2020). The Republic of Indonesia joint external monitoring Mission for tuberculosis. Indonesia: Ministry of Health Republic of Indonesia.

[ref16] Ministry of Health Republic Indonesia. Dashboard data kondisi TB di Indonesia. (2023). Available at: https://tbindonesia.or.id/pustaka-tbc/dashboard/. (Accessed on November 9, 2023).

[ref17] Ministry of Health Republic of Indonesia. Petunjuk teknis dan pemantapan mutu: pemeriksaan biakan, identifikasi, dan uji kepekaan *Mycobacterium tuberculosis* complex terhadap obat anti Tuberkulosis pada media padat dan media cair. Indonesia: Ministry of Health of the Republic of Indonesia. (2021).

[ref18] Noordhoek G. T. Kolk A. Bjune G. Catty D. Dale J. W. Fine P.. (1994). Sensitivity and specificity of PCR for detection of *Mycobacterium tuberculosis*: a blind comparison study among seven laboratories. J. Clin. Microbiol. 32, 277–284. doi: 10.1128/jcm.32.2.277-284.1994 8150935 PMC263025

[ref19] Peng J. Yu X. Cui Z. Xue W. Luo Z. Wen Z.. (2017). Multi-fluorescence real-time PCR assay for detection of RIF and INH resistance of *M.Tuberculosis*. Front. Microbiol. 7:7618. doi: 10.3389/fmicb.2016.00618, PMID: 27199947 PMC4850356

[ref20] Ramaswamy S. Musser J. M. (1998). Molecular genetic basis of antimicrobial agent resistance in *Mycobacterium tuberculosis*: 1998 update. Tuber. Lung Dis. 79, 3–29. doi: 10.1054/tuld.1998.0002 10645439

[ref21] Sarmiento O. L. Weigle K. A. Alexander J. Weber D. J. Miller W. C. (2003). Assessment by meta-analysis of PCR for diagnosis of smear-negative pulmonary tuberculosis. J. Clin. Microbiol. 41, 3233–3240. doi: 10.1128/JCM.41.7.3233-3240.2003, PMID: 12843069 PMC165327

[ref22] Svensson E. Folkvardsen D. B. Rasmussen E. M. Lillebaek T. (2021). Detection of *Mycobacterium tuberculosis* complex in pulmonary and extrapulmonary samples with the FluoroType MTBDR assay. Clin. Microbiol. Infect. 27, 1514.e1–1514.e4. doi: 10.1016/j.cmi.2020.12.020, PMID: 33421581

[ref23] Tan M. F. Ng W. C. Chan S. H. Tan W. C. (1997). Comparative usefulness of PCR in the detection of *Mycobacterium tuberculosis* in different clinical specimens. J. Med. Microbiol. 46, 164–169. doi: 10.1099/00222615-46-2-164, PMID: 9060877

[ref24] Vinuesa V. Borras R. Brionnes M. L. Clari M. A. Cresencio V. Gimenez E.. (2018). Performance of highly sensitive *Mycobacterium tuberculosis* complex real-time PCR assay for diagnosis of pulmonary tuberculosis in a low-prevalence setting: a prospective intervention study. J. Clin. Microbiol. 56, e00116–e00118. doi: 10.1128/JCM.00116-18, PMID: 29540457 PMC5925698

[ref25] Wang M. G. Xue M. Wu S. Q. Zhang M. M. Wang Y. Liu Q.. (2019). Abbott RealTime MTB and MTB RIF/INH assays for the diagnosis of tuberculosis and rifampicin/isoniazid resistance. Infect. Genet. Evol. 71, 54–59. doi: 10.1016/j.meegid.2019.03.012, PMID: 30902741

[ref26] World Health Organization (2023). Global tuberculosis report. Geneva: WHO.

