# Supplementary material for: Evaluation of a real-time PCR assay performance to detect Mycobacterium tuberculosis, rifampicin, and isoniazid resistance in sputum specimens: a multicenter study in two major cities of Indonesia
Source: Front Microbiol. 2024 May 10;15:1372647. doi: 10.3389/fmicb.2024.1372647 (PMC11123600; doi:10.3389/fmicb.2024.1372647)
Supplement: Supplementary file 1 [file Table_1.pdf]

Supplementary Material

Supplementary Table 1. List of Indigen MTB/DR TB RT PCR and Sequencing primer

qPCR Primer

Primer	Target	Sequence (5'-3')
F-IS6110	IS6110	GGATAACGTCTTTCAGGTCGAG
R-IS6110	IS6110	TATGACAATGCACTAGCCGAG
F-Lambda	Lambda	CGATAGACCTTACAGTGCT
R-Lambda	Lambda	CTTACCCCAACCAACAGG
F-rpoB	rpoB	CGGCGGTCTGTCACGTGAG
R-rpoB	rpoB	TCCTTGATCGCGGCGACC
F-katG	katG	TTCCAGCCCAAGCCCATC
R-katG	katG	TTCCTCGAGATCCTGTACGGCT
F-inhA	inhA	GTAACGTGGCTGCGATTTCC
R-inhA	inhA	TGACTGCCACAGCCACTGAA

Sequencing Primer

Primer	Target	Sequence (5'-3')
F-rpoB	rpoB	CGGCGGTCTGTCACGTGAG
R-rpoB	rpoB	TCCTTGATCGCGGCGACC
