# Supplementary material for: Evaluation of a real-time PCR assay performance to detect Mycobacterium tuberculosis, rifampicin, and isoniazid resistance in sputum specimens: a multicenter study in two major cities of Indonesia
Source: Front Microbiol. 2024 May 10;15:1372647. doi: 10.3389/fmicb.2024.1372647 (PMC11123600; doi:10.3389/fmicb.2024.1372647)
Supplement: Supplementary file 2 [file Data_Sheet_1.pdf]

Supplementary Material

Supplementary Data 1. Table of Indigen MTB/DR TB RT PCR LoD test results

Detection of *M. tuberculosis*

Target Concentration (CFU/mL)	Number of Replicates Tested	Number of Replicates Detected	Rate of Detection
**400**	**20**	**20**	**100%**
200	20	18	90%
100	20	16	80%
50	20	8	40%
10	20	2	10%

Detection of *M. tuberculosis* with Rifampicin resistance (rpoB S531L)

Target Concentration (CFU/mL)	Number of Replicates Tested	Number of Replicates Detected	Rate of Detection
10,000	20	20	100%
8,000	20	20	100%
**6,000**	**20**	**19**	**95%**
4,000	20	1	5%
2,000	20	0	00%

Detection of *M. tuberculosis* with Rifampicin resistance (rpoB H526Y)

Target Concentration (CFU/mL)	Number of Replicates Tested	Number of Replicates Detected	Rate of Detection
10,000	20	20	100%
8,000	20	20	100%
**6,000**	**20**	**19**	**95%**
4,000	20	12	60%
2,000	20	8	40%

Detection of *M. tuberculosis* with Isoniazid resistance (katG S315T)

Target Concentration (CFU/mL)	Number of Replicates Tested	Number of Replicates Detected	Rate of Detection

1,000	20	20	100%
800	20	20	100%
600	20	20	100%
**400**	**20**	**19**	**95%**
200	20	15	75%
**Detection of *M. tuberculosis* with Isoniazid resistance (inhA -C15T)**			
8,000	20	20	100%
**6,000**	**20**	**20**	**100%**
4,000	20	6	30%
2,000	20	3	20%
1,000	20	2	10%
Detection of M. smegmatis (NTM)			
**Target Concentration (CFU/mL)**	**Number of Replicates Tested**	**Number of Replicates Detected**	**Rate of Detection**
100,000	20	20	100%
**50,000**	**20**	**19**	**95%**
40,000	20	11	55%
20,000	20	3	15%
