# Supplementary material for: Evaluation of a real-time PCR assay performance to detect Mycobacterium tuberculosis, rifampicin, and isoniazid resistance in sputum specimens: a multicenter study in two major cities of Indonesia
Source: Front Microbiol. 2024 May 10;15:1372647. doi: 10.3389/fmicb.2024.1372647 (PMC11123600; doi:10.3389/fmicb.2024.1372647)
Supplement: Supplementary file 3 [file Data_Sheet_2.pdf]

Supplementary Material

Supplementary Data 2. Table summary of Indigen MTB/DR TB RT-PCR in-silico cross reactivity analysis

No.	Species	Accession No.	*IS6110* Probe	
			% Homology	≥ 80% Homology? (Yes/No)
Mycobacteria				
1	*Mycobacterium abscessus*	CP060408.1	58%	No
2	*Mycobacterium asiaticum*	GU362430.1	54%	No
3	*Mycobacterium avium* subsp. *avium*	CP046507.1	58%	No
4	*Mycobacterium bohemicum*	HQ235059.1	54%	No
5	*Mycobacterium celatum*	AF312688.1	67%	No
6	*Mycobacterium chelonae*	CP034383.1	54%	No
7	*Mycobacterium flavescens*	LR134353.1	46%	No
8	*Mycobacterium fortuitum*	AP025518.1	21%	No
9	*Mycobacterium gastri*	JX215335.1	58%	No
10	*Mycobacterium genavense*	LC533965.1	54%	No
11	*Mycobacterium goodii*	CP012150.1	42%	No
12	*Mycobacterium gordonae*	CP070973.1	58%	No
13	*Mycobacterium haemophilum*	CP011883.2	42%	No
14	*Mycobacterium immunogenum*	CP016189.1	67%	No
15	*Mycobacterium intracellulare*	CP023149.1	50%	No
16	*Mycobacterium kansasii*	CP089218.1	58%	No
17	*Mycobacterium kumamotonense*	MK890454.1	42%	No
18	*Mycobacterium lentiflavum*	CP092423.1	33%	No
19	*Mycobacterium leprae*	CP029543.1	58%	No
20	*Mycobacterium malmoense*	CP080999.1	46%	No
21	*Mycobacterium marinum*	CP024190.1	54%	No

22	*Mycobacterium massiliense*	AP018436.1	58%	No
23	*Mycobacterium mucogenicum*	CP062008.1	50%	No
24	*Mycobacterium nonchromogenicum*	MN049930.1	54%	No
25	*Mycobacterium phlei*	LR134347.1	54%	No
26	*Mycobacterium scrofulaceum*	LC082326.1	54%	No
27	*Mycobacterium shimoidei*	AB292553.1	50%	No
28	*Mycobacterium simiae*	AP022568.1	50%	No
29	*Mycobacterium smegmatis*	CP027541.1	33%	No
30	*Mycobacterium szulgai*	NR_118584.1	54%	No
31	*Mycobacterium terrae*	AP022564.1	38%	No
32	*Mycobacterium thermoresistibile*	LT906483.1	50%	No
33	*Mycobacterium triviale*	KT020747.1	54%	No
34	*Mycobacterium ulcerans*	LR135168.1	54%	No
35	*Mycobacterium xenopi*	AP022314.1	58%	No
Fungi				
1	*Aspergillus fumigatus*	CP084980.1	46%	No
2	*Blastomyces dermatitidis*	XM_045418537.1	54%	No
3	*Candida albicans*	CP032019.1	25%	No
4	*Candida glabrata*	CR380952.1	50%	No
5	*Candida krusei*	CP039612.1	29%	No
6	*Candida parapsilosis*	HE605208.1	54%	No
7	*Candida tropicalis*	CP047872.1	21%	No
8	*Cryptococcus neoformans*	CP047906.1	54%	No
9	*Histoplasma capsulatum*	CP069114.1	50%	No
10	*Penicillium* spp.	MW690135.1	50%	No
11	*Rhizopus* spp.	MG872962.1	50%	No
12	*Scedosporium* spp.	XM_016785468.1	50%	No
Viruses				
1	Adenovirus	OP270254.1	46%	No
2	HIV	ON500892.1	50%	No

3	Human Influenza Virus Type A	CY112253.1	46%	No
4	Human Influenza Virus Type B	MN589446.1	46%	No
5	Human Metapneumovirus	MK588635.1	29%	No
6	Human Parainfluenza Virus Type 1	AF457102.1	54%	No
7	Human Parainfluenza Virus Type 2	JF912194.1	58%	No
8	Human Parainfluenza Virus Type 3	KF687354.1	54%	No
9	Human Parainfluenza Virus Type 4	KF878965.2	54%	No
10	Mumps Virus	MH426702.1	42%	No
11	Respiratory Syncytial Virus Rhinovirus	OP320398.1	54%	No
12	Rubella Virus	MH745081.1	33%	No
13	Rubeola Virus	ON035936.1	58%	No
14	Varicella Zoster Virus	MH709377.1	54%	No
Bacteria				
1	*Acinetobacter baumannii*	CP050385.1	50%	No
2	*Acinetobacter calcoaceticus*	CP020000.1	50%	No
3	*Actinomyces israelii*	LR134357.1	25%	No
4	*Bacillus cereus*	CP072769.1	46%	No
5	*Bacillus subtilis*	CP035403.1	54%	No
6	*Bacteroides fragilis*	CP054003.1	54%	No
7	*Burkholderia cepacia*	CP073638.1	50%	No
8	*Chlamydia pneumoniae*	LN847257.1	58%	No
9	*Citrobacter freundii*	CP085726.1	42%	No
10	*Clostridium* spp.	CP119188.1	58%	No
11	*Corynebacterium diphtheriae*	CP038504.1	33%	No
12	*Corynebacterium jeikeium*	CP033784.1	46%	No
13	*Corynebacterium pseudodiptheriticum*	CP091863.1	46%	No
14	*Eikenella corrodens*	CP034670.1	63%	No
15	*Enterobacter aerogenes*	CP094272.1	50%	No
16	*Enterobacter cloacae*	CP071861.1	42%	No
17	*Enterococcus faecalis*	CP022312.1	38%	No

18	*Enterococcus faecium*	AP026666.1	54%	No
19	*Escherichia coli*	AP026510.1	58%	No
20	*Fusobacterium nucleatum* subsp. *polymorphum*	CP021934.1	50%	No
21	*Haemophilus influenzae*	CP031250.1	50%	No
22	*Haemophilus parahaemolyticus*	CP038817.1	50%	No
23	*Haemophilus parainfluenzae*	FQ312002.1	38%	No
24	*Kingella kingae*	LN869922.1	63%	No
25	*Klebsiella oxytoca*	CP089399.1	38%	No
26	*Klebsiella pneumoniae*	CP077814.1	50%	No
27	*Lactobacillus acidophilus*	CP054559.1	46%	No
28	*Legionella micdadei*	CP020615.1	58%	No
29	*Legionella pneumophila*	CP040987.1	50%	No
30	*Leuconostoc* spp.	CP028255.1	58%	No
31	*Listeria monocytogenes*	CP053630.1	63%	No
32	*Moraxella catarrhalis*	EU401938.1	50%	No
33	*Mycoplasma pneumoniae*	CP039761.1	63%	No
34	*Neisseria gonorrhoeae*	CP032429.2	38%	No
35	*Neisseria lactamica*	LR590477.1	54%	No
36	*Neisseria meningitidis*	CP031329.1	29%	No
37	*Neisseria mucosa*	CP053939.1	29%	No
38	*Neisseria sicca*	CP072524.1	58%	No
39	*Nocardia brasiliensis*	CP046171.1	50%	No
40	*Nocardia farcinica*	CP031418.1	42%	No
41	*Nocardia otitidiscaviarum*	CP041695.1	54%	No
42	*Pediococcus* spp.	CP053421.1	29%	No
43	*Peptostreptococcus* spp.	FJ410395.1	63%	No
44	*Proteus mirabilis*	CP046048.1	58%	No
45	*Proteus vulgaris*	CP054157.1	50%	No
46	*Pseudomonas aeruginosa*	CP101912.1	46%	No
47	*Rhodococcus equi*	CP118697.1	46%	No
48	*Serratia marcescens*	CP041131.1	38%	No
49	*Staphylococcus aureus*	CP062443.1	54%	No
50	*Staphylococcus epidermidis*	CP093222.1	33%	No
51	*Staphylococcus haemolyticus*	CP052055.1	42%	No

52	*Staphylococcus lugdunensis*	AP021848.1	50%	No
53	*Stenotrophomonas maltophilia*	CP060259.1	54%	No
54	*Streptococcus agalactiae*	CP049938.1	42%	No
55	*Streptococcus anginosus*	CP012719.1	54%	No
56	*Streptococcus equi*	CP046042.2	42%	No
57	*Streptococcus mitis*	CP047883.1	46%	No
58	*Streptococcus mutans*	CP050273.1	50%	No
59	*Streptococcus pneumoniae*	CP036529.1	42%	No
60	*Streptococcus pyogenes*	CP043530.1	50%	No
61	*Streptococcus salivarius*	CP018187.1	42%	No
62	*Streptococcus sanguinis*	LR134002.1	58%	No
63	*Streptomyces anulatus*	CP086102.1	21%	No
64	*Tsukamurella* spp.	CP019066.1	67%	No
65	*Veillonella parvula*	CP019721.1	21%	No
66	*Yersinia enterocolitica*	CP107100.1	50%	No
